# The Role of Autoimmune Diseases in the Development of Aortic Aneurysms

**DOI:** 10.31083/RCM50176

**Published:** 2026-09-04

**Authors:** Jaroslav Michalek, Martin Petrek, Jana Petrkova

**Affiliations:** ^1^Department of Clinical and Molecular Pathology, University Hospital Olomouc, 77900 Olomouc, Czech Republic; ^2^Department of Pathological Physiology, Faculty of Medicine and Dentistry, Palacky University Olomouc, 77900 Olomouc, Czech Republic; ^3^Department of Internal Medicine I - Cardiology, University Hospital Olomouc, 77900 Olomouc, Czech Republic

**Keywords:** aortic aneurysm, sudden cardiac death, risk factors, autoimmune diseases, diagnosis, therapeutics

## Abstract

Aortic aneurysms are abnormal dilations of the aortic wall and represent a significant cause of morbidity and mortality worldwide. In many cases, especially when the thoracic aorta is involved, the first and only symptom is sudden death. While aortic aneurysms are traditionally associated with factors such as advanced age, hypertension, and atherosclerosis, growing evidence points to a critical role for autoimmune diseases in their pathogenesis. The hallmarks of autoimmune disorders, such as chronic systemic inflammation, immune dysregulation, and vascular tissue remodeling, can create a pro-aneurysmal environment within the vasculature. This review explores the complex interplay between autoimmune diseases and aortic aneurysm formation, focusing on pathogenic mechanisms, specific disease associations, and implications for diagnosis and management. A better understanding of these relationships is vital for advancing therapeutic strategies aimed at mitigating vascular complications in affected patients.

## 1. Introduction

Autoimmune diseases are characterized by a loss of immunological tolerance, leading to self-reactive immune responses via multiple pathways [1,2]. This has profound systemic implications, often extending beyond the traditionally affected tissues or organs. Recent research has shed light on the role of these disorders in the development and progression of vascular pathologies, particularly aortic aneurysms (AAs) [3]. AAs are abnormal dilations of the aorta that pose significant clinical challenges due to their potential for rupture and associated fatal outcomes [4,5]. Established risk factors such as smoking, hypertension, and genetic predispositions (e.g., connective tissue disorders) remain critical in understanding the pathogenesis of aneurysms. However, the contribution of chronic inflammation and immune-mediated vascular damage also warrants further exploration [6,7,8].

Autoimmune diseases such as systemic lupus erythematosus (SLE), rheumatoid arthritis (RA), Behçet’s disease, and IgG4-related disease are increasingly recognized as conditions that predispose patients to vascular complications, including AAs. These diseases exhibit shared pathophysiological hallmarks that weaken vessel integrity over time, including persistent inflammation, immune cell infiltration into vascular structures, and dysregulated tissue remodeling [9,10]. A better understanding of the intersection between autoimmune mechanisms and aortic pathology may help clinicians and researchers to better identify at-risk populations and develop targeted interventions to improve patient outcomes.

In this review, we dissect the complex relationship between autoimmune diseases and AAs by examining their epidemiology, underlying pathogenetic mechanisms, diagnostic challenges, and therapeutic implications. Furthermore, we highlight specific autoimmune conditions that are frequently associated with an increased risk of AAs.

Search Strategy and Information Sources: A comprehensive literature search was conducted across PubMed and Google Scholar, from database inception through to 2026. The search strategy was developed by combining Medical Subject Headings (MeSH) and relevant free-text keywords using Boolean operators (AND, OR). The key concepts were “autoimmune diseases” (including specific diseases like systemic lupus erythematosus, rheumatoid arthritis, Behçet’s disease, and IgG4-related disease) and “aortic aneurysms”. No language restrictions were applied. To ensure literature saturation, the reference lists of all included studies and relevant prior systematic reviews were manually screened.

## 2. Overview of Aortic Aneurysms

### 2.1 Definition and Pathophysiology

An AA is defined as an abnormal localized dilation of the aorta exceeding 50% of its normal diameter. Based on their anatomical location, aneurysms are classified as either thoracic (thoracic aortic aneurysm, TAA) or abdominal (abdominal aortic aneurysm, AAA) [11]. This reflects differences in embryological origin, mechanical stressors, and molecular influences affecting different segments of the aorta. The progression of an aneurysm is driven by complex pathophysiological processes that compromise structural integrity within the arterial wall.

The arterial wall consists of three distinct layers: the intima (innermost layer), media (middle muscular layer), and adventitia (outer supportive layer). The development of an AA involves pathological changes across these layers. Key features include elastin degradation within the media by matrix metalloproteinases (MMPs), dysregulation of collagen turnover leading to weakened structural support, infiltration by inflammatory cells such as macrophages and lymphocytes, oxidative stress-induced damage to endothelial cells, and reduced smooth muscle cell viability. Together, these processes culminate in thinning of the vessel wall and dilation under physiological pressure [12,13,14].

Hemodynamic stress plays an instrumental role in initiating these pathological changes. Regions subjected to high mechanical load—such as the infrarenal segment in AAA and the ascending thoracic region in TAA—are particularly vulnerable. While established risk factors like hypertension can amplify the hemodynamic strain on these regions, autoimmune-related immune dysregulation introduces additional complexity through sustained inflammation that accelerates tissue degradation [15,16]. The known risk factors for AAs are summarized in Table 1.

**Table 1.** **Risk factors for aortic aneurysms**.

Risk factor	Description/Mechanism
Age	Risk increases with age, particularly over 60 years
Male sex	Higher incidence and risk of rupture in males
Family history	First-degree relatives have 2–4–fold higher risk
Genetic disorder	Marfan syndrome, Ehlers-Danlos syndrome, Loeys-Dietz syndrome
Smoking	Strongest modifiable factor; promotes inflammation and matrix degradation
Hypertension	Increases hemodynamic stress on the aortic wall
Hyperlipidemia	Contributes to atherosclerosis and vascular wall damage
Atherosclerosis	Common etiologic factor, especially in AAA
Obesity	Associated with chronic inflammation and metabolic strain
Physical inactivity	Indirectly increases cardiovascular and aneurysm risk
Chronic systemic inflammation	Occurs in autoimmune diseases (SLE, RA, vasculitis); leads to VSMC damage and wall thinning
Elevated cytokine levels (e.g., TNF-α, IL-6)	Stimulate MMPs, endothelial dysfunction, and vessel wall remodeling
Elevated CRP	Marker of inflammation; associated with aneurysm progression
Syphilitic aortitis	Historically significant cause of TAA
Mycotic aneurysms	Arise from bacterial or fungal infection of the arterial wall
Trauma or prior vascular surgery	Localized injury weakens vessel integrity

TNF-α, tumor necrosis factor-alpha; IL-6, interleukin-6; CRP, C-reactive protein; AAA, abdominal aortic aneurysm; SLE, systemic lupus erythematosus; RA, rheumatoid arthritis; VSMC, vascular smooth muscle cell; MMPs, matrix metalloproteinases; TAA, thoracic aortic aneurysm.

### 2.2 Epidemiological Aspects

AAs are significant contributors to global morbidity and mortality due to vascular disease. The main complication of AA is rupture, which can lead to uncontrolled bleeding. This results in the body going into shock and potentially to cardiac arrest and death. In many cases, the first and only symptom of an AA is sudden death, particularly if the aneurysm is located in the thoracic aorta [17]. Because AAs can be asymptomatic, early detection through screening and awareness of symptoms like chest or back pain, shortness of breath, and loss of consciousness is crucial for timely intervention and the potential prevention of sudden death. Advances in diagnostic imaging have facilitated early detection in asymptomatic individuals, but incidence rates remain high due to aging populations worldwide [18]. Epidemiological patterns vary according to the location: AAAs are more prevalent than TAAs in the general population, but show distinct demographic distributions.

AAAs primarily affect older males with long-standing exposure to lifestyle risk factors such as smoking or hyperlipidemia. However, the incidence of AAA is increasing among females with underlying inflammatory disorders or connective tissue abnormalities. Conversely, TAAs exhibit broader etiological diversity and encompass genetic syndromes like Marfan syndrome and Ehlers-Danlos syndrome, alongside secondary causes involving vasculitis-associated autoimmune disorders [15,16,19].

Autoimmune diseases can significantly alter this epidemiological landscape by introducing unique patient subsets with higher susceptibility, in the absence of traditional risk factors such as advanced age or smoking history. Female predominance within autoimmune conditions further compounds this effect, since conventional male-dominated AAA cohorts typically underrepresent women who are at elevated vascular risk due solely to their underlying systemic illness rather than modifiable external influences.

The burden imposed by autoimmune-driven vascular complications necessitates dedicated research efforts. These should investigate tailored surveillance programs targeting vulnerable patient populations, while refining preventive strategies aimed at mitigating risks specific to chronic inflammatory processes [20].

## 3. Autoimmune Diseases and Their Characteristics

### Immune System Dysregulation in Autoimmune Disorders

Autoimmune diseases arise from disruptions in immune tolerance mechanisms that normally prevent self-reactivity. Pathways involved in autoimmunity are often multifaceted and combined, with most autoimmune diseases having a genetic component [2].

Central tolerance within the thymus eliminates autoreactive T cells during early development, whereas peripheral tolerance mechanisms suppress any residual self-reactive lymphocytes through regulatory T cells (Tregs) or inhibitory signals mediated by checkpoint molecules such as Cytotoxic T-lymphocyte associated protein 4 (CTLA-4) or /Programmed cell death protein 1 (PD-1)/Programmed death-ligand 1 (PD-L1) pathways [21].

Failures within these systems result in the aberrant activation of both innate (e.g., macrophages) and adaptive (e.g., autoreactive B/T cells) immune components against self-antigens present within tissues or organs, including vascular structures like the aorta. Chronic activation leads to sustained cytokine production (e.g., IL-17A via T-helper 17 (Th17) cells) that perpetuates inflammation. Alongside this inflammatory cascade are antibody-mediated effects seen prominently among autoantibody-positive disorders, such as SLE or Sjögren’s syndrome [1,22,23].

## 4. Pathogenic Mechanisms Linking Autoimmune Diseases to Aortic Aneurysms

### 4.1 Chronic Inflammation and Vascular Damage

Chronic inflammation is a hallmark of autoimmune diseases and serves as a critical mechanism in the pathogenesis of AAs. Persistent immune activation leads to the recruitment of inflammatory cells such as macrophages, lymphocytes, and neutrophils to the vascular wall. These infiltrating immune cells release pro-inflammatory cytokines (e.g., tumor necrosis factor-alpha [TNF-α], interleukin-6 [IL-6]), chemokines, and reactive oxygen species (ROS) that exacerbate endothelial dysfunction and damage vascular smooth muscle cells (VSMCs). Over time, this inflammatory milieu weakens the structural integrity of the aortic wall.

The role of proinflammatory cytokines (IL-6, IL-10, IL-17, TNF-α) and chemokines (IL-8, CCL1, CCL5) as crucial regulators of inflammation in the development of AAs was initially suggested by studies of individual cytokine proteins, and later confirmed using protein multiplex assays [24,25,26,27]. The same cytokines/chemokines have also been implicated in the pathogenesis and progression of autoimmune diseases, particularly SLE and RA [28,29,30,31,32,33]. Recent studies employing methodologies such as single-cell sequencing and Mendelian randomization have revealed additional candidate proteins relevant for AAs, such as the chemokine CXCL10 and the fibroblast growth factor FGF5 [34]. Importantly, CXCL10 has also been implicated in autoimmune diseases, namely SLE [35].

A key molecular pathway implicated in the process of vascular damage involves MMPs, particularly MMP-2 and MMP-9, which are upregulated in response to inflammation. These proteolytic enzymes degrade extracellular matrix (ECM) components such as elastin and collagen within the medial layer of the aorta. The resulting loss of elastin fibers undermines the vessel’s ability to withstand hemodynamic pressures, predisposing it to dilation and eventually the formation of aneurysms. Patients with autoimmune diseases such as SLE exhibit elevated MMP activity in their vasculature compared to healthy controls.

Additionally, endothelial cells exposed to chronic inflammation show increased permeability and reduced nitric oxide production, impairing their protective function against mechanical stress and thrombosis. In autoimmune conditions such as RA and Behçet’s disease, immune-mediated endothelial injury is further compounded by immune complex deposition and complement activation. This sustained assault on the endothelium contributes to maladaptive vascular remodeling that favors the development of aneurysms [36,37,38].

### 4.2 Immune-Mediated Tissue Remodeling and Vessel Weakening

Autoimmune diseases often involve aberrant tissue repair processes driven by dysregulated immune responses. In the context of AAs, immune-mediated tissue remodeling plays a pivotal role in weakening the vessel wall. In particular, T-helper 1 (Th1) and Th17 cells are implicated in promoting a pro-inflammatory environment through interferon-gamma (IFN-γ) and interleukin-17 (IL-17), respectively. These cytokines stimulate fibroblasts, macrophages, and VSMCs to secrete additional degradative enzymes, while inhibiting normal repair mechanisms.

Furthermore, the generation of autoantibodies in diseases such as SLE or systemic sclerosis can directly target vascular structures or cross-react with shared antigens on connective tissue components. For instance, antiphospholipid antibodies associated with antiphospholipid syndrome contribute to arterial wall thrombosis, ischemia, and subsequent remodeling through upregulation of tissue factor expression on endothelial cells.

The maladaptive repair process also involves excessive collagen deposition by myofibroblasts in response to chronic inflammation. However, this newly formed collagen is often disorganized and lacks the tensile strength required for adequate structural support. Combined with elastin degradation, these changes create an environment that is primed for mechanical failure under hemodynamic stress.

Lastly, adventitial inflammation—a characteristic feature of autoimmune-related vasculitides—can further contribute to vessel weakening. Infiltrating immune cells in the adventitia produce angiogenic factors such as vascular endothelial growth factor (VEGF), thereby promoting neovascularization that paradoxically exacerbates local hypoxia and oxidative stress within the arterial wall [39,40,41].

The manifestation of the above-described alterations can be assessed at the microscopic level by histopathology. Non-specific findings range from chronic inflammatory infiltrates and immune complex depositions to collagen degradation and matrix disorganization, intimal hyperplasia and endothelial damage, eventually to vasculitis pattern and/or thrombus formation [42,43].

Schematic representation of key mechanisms implicated in pathogenesis of AAs is depicted in Fig. 1. Some of these processes are operating in AAs due to other, general risk factors and/or co-existing diseases, the role of autoimmunity as an initiating trigger is emphasized in the right upper corner.

**Fig. 1.**
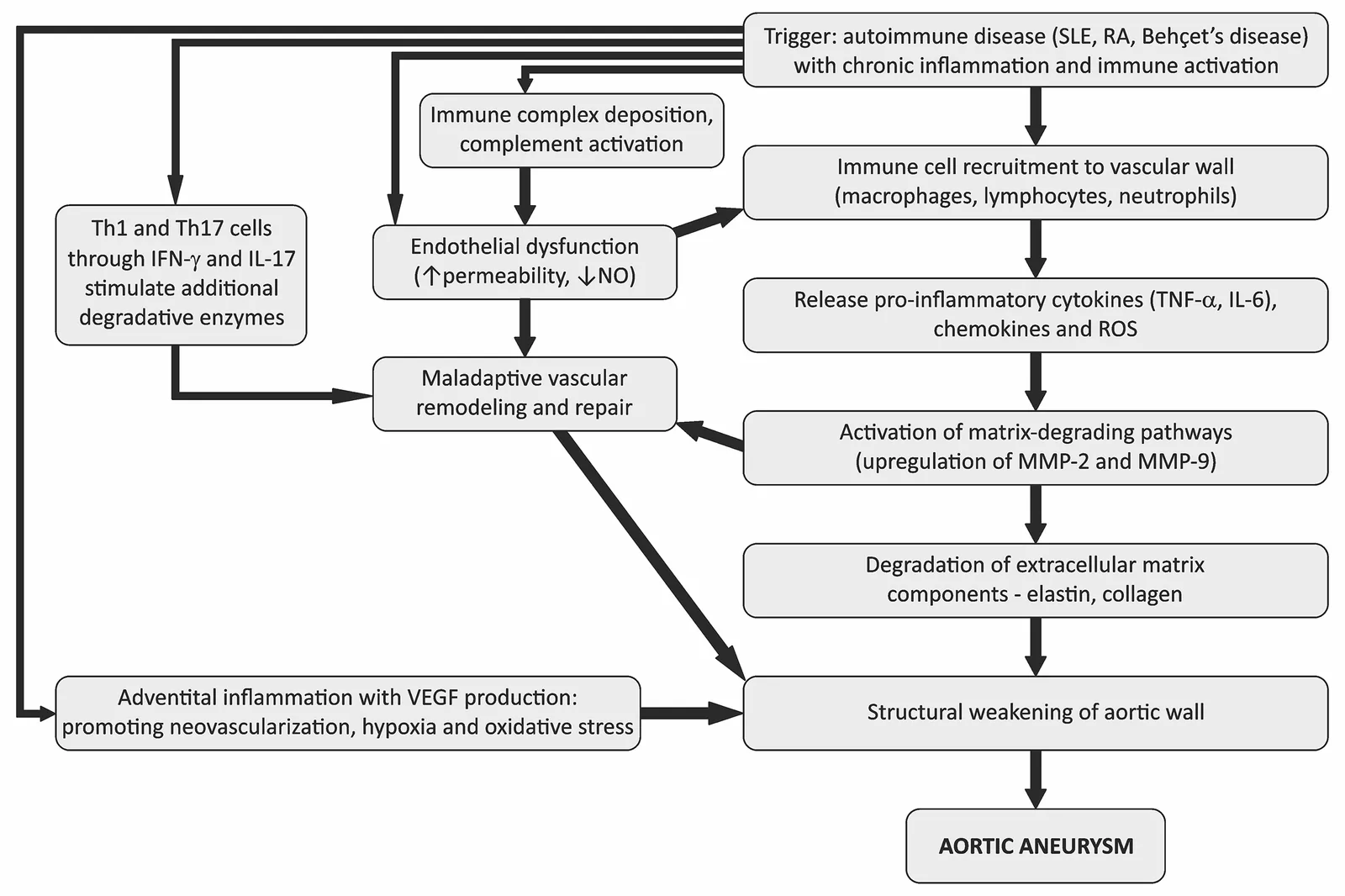
**The key pathogenetic mechanisms implicated in aortic aneurysms in the context of autoimmune diseases**. IFN-γ, interferon-gamma; IL-6, interleukin-6; IL-17, interleukin-17; MMP-2, matrix metalloproteinase-2; MMP-9, matrix metalloproteinase-9; NO, nitric oxide; RA, rheumatoid arthritis; ROS, reactive oxygen species; SLE, systemic lupus erythematosus; Th1, T-helper 1 cells; Th17, T-helper 17 cells; TNF-α, tumor necrosis factor-alpha; VEGF, vascular endothelial growth factor.

### 4.3 Genetic Susceptibility and Overlap Between Conditions

Genetic predisposition plays a crucial role in determining both autoimmune susceptibility and vulnerability to AAs. Shared genetic loci between autoimmune diseases and vascular disorders suggest that common molecular pathways underpin these conditions. For example, polymorphisms in the human leukocyte antigen (HLA) genes have been associated with both SLE and vasculitis-related complications [44,45] such as AAs [41]. While these reports suggest a relationship between AAs and specific HLA alleles, the relatively small cohort size [46] and methodical constraints have led to inconsistent and irreproducible results [47]. The advent of new methodical concepts and technological progress, such as next-generation sequencing [48], now provide opportunities to examine plausible contributions of HLA variations to the development of AAs.

In addition to HLA variants, specific single nucleotide polymorphisms (SNPs) in genes with immune response and regulatory functions—such as cytokines, their receptors, or polymorphisms affecting toll-like receptor signaling [49]—may increase susceptibility to vascular damage among individuals with autoimmune diseases. For instance, SNP variations in genes encoding IL-6 receptors or TNF-α pathways could amplify inflammatory responses at arterial sites that are predisposed to aneurysm formation. For some AA-associated SNPs, correlations with altered protein levels in the circulation were observed, suggesting their plausible clinical relevance [27]. Verification of these experimental findings is a prerequisite for future translational applications as potential diagnostic tools or targets for therapy, and thus represents an area for future research [50,51]. In this context, valuable additional information can be obtained from Genome-wide association studies (GWAS)—large-scale analyses across entire genome. The last five years have brought several reports from GWAS or their meta-analyses in the context of AA [52,53,54,55]. The identified candidate gene loci, the encoded proteins, and their corresponding functional pathways can be linked to inflammation, ECM degradation, vascular development and remodeling, altered lipid/cholesterol metabolism, and atherogenesis [53,54], which constitute major processes involved in AA pathobiology. The prominent examples include CD40/CD40 ligand (CD40L) and low-density lipoprotein receptor-related protein 1 (LRP1) [54], as well as several loci that affect lipid metabolism, indicating a possible connection with proprotein convertase subtilisin/kexin type 9 (PCSK9) as a therapeutic target for AA [53].

Regarding the autoimmune component in the AA pathogenesis, it is worth mentioning that some of the AA GWAS nominees were pathophysiologically relevant, also for autoimmune diseases – for example, the already mentioned CD40/CD40L [54] plays a regulatory role in autoimmunity [56,57]. Vice versa, IL-23/IL-23R and IL-10, which have been previously implicated in AA pathomechanisms [58,59], were recently nominated as candidates by an *in silico* study investigating variants common across 15 autoimmune diseases [60]. Plausibly, genetic architecture and regulatory mechanisms are, at least in part, shared between AA and autoimmunity.

Genetically determined connective tissue disorders such as Marfan syndrome (MFS) or Loeys-Dietz syndrome exhibit phenotypic overlap with certain autoimmune conditions due to shared alterations affecting ECM proteins such as fibrillin-1 or transforming growth factor-beta (TGF-β). Although these syndromes are not classical autoimmune diseases, they demonstrate how genetic abnormalities affecting vascular integrity can intersect with immunological processes during the pathogenesis of aneurysms. In this context, a recent study implicated fibroblast growth factor 12 (FGF12) as a critical regulator of aberrant mechanosignaling in aortic VSMCs and as a key contributor to aneurysm formation in MFS [61].

### 4.4 Role of Epigenetic Mechanisms

Epigenetic modifications driven by chronic inflammation may further exacerbate susceptibility to AAs among patients with autoimmune diseases. Histone acetylation or deoxyribonucleic acid (DNA) methylation changes induced by inflammatory cytokines can alter gene expression patterns linked to collagen synthesis or elastin degradation pathways within the aortic wall [37,41]. Other post-transcriptional modifications implicated in autoimmunity, such as RNA methylation [62], may also play a role in the development of AAs. Increased mRNA methylation of adenosine was reported in the AA patients [63], and was also recently documented in a mouse model [64]. Since these post-transcriptional factors operate both in autoimmunity and in AA development and progression, this provides more evidence, albeit indirect, of a shared pathological mechanism. Master regulation of gene expression for key immune, inflammatory, and/or remodeling mediators and receptors can thus be proposed as an additional core mechanism contributing to the development of AAs in autoimmune diseases.

microRNAs (miRNAs) represent another mechanism for regulating the expression of genes involved in immune reactions and their corresponding alterations in autoimmune disease [62]. These small, non-coding RNAs were previously reported in connection with altered cardiovascular vasculature (e.g., [65]). Indeed, investigators have reported a plausible role for specific miRNAs in AA, such as miR-15a-5p [66]. miRNA research in the field of AAs may have a translational impact due to the potential therapeutic application of miRNA inhibition or silencing. In this regard, Winski et al. [66] reported a beneficial effect of miR-15a-5p inhibition on AA. Although this was observed in a mouse model, it forms part of ongoing research of miRNA-based interventions as a AA new therapeutic option [67].

Similar to GWAS, large-scale studies at the level of epigenome are represented by EWAS (Epigenome Wide Association Studies), which instead on sequence variants focus on DNA methylation alterations and other changes across epigenome. Searching the current version of the Epigenome Atlas [68], there has been no record related to AAs. Most recently, Yuan et al. [69] reported data from EWAS in 1324 AAA patients compared with 42,065 controls. In this study, archived in medRxiv, methylation markers were associated with cardiometabolic and immune/inflammatory pathways [69]. While these findings are consistent with the pathogenetic role of inflammation and metabolic processes in AA, future EWAS investigations in context of AAs are necessary for data confirmation and extension. In our opinion, to achieve eventual translational relevance of these perspective studies, the complex spectrum of processes occurring at the (epi) genetic level during AA origin, development and progression should be complemented by other omics approaches, e.g., proteomics, lipidomics and/or metabolomics, see e.g., [70].

## 5. Specific Autoimmune Diseases Are Associated With an Increased Risk of Aortic Aneurysms

### 5.1 Systemic Lupus Erythematosus and Aneurysm Formation

SLE is an archetypal multisystem autoimmune disease characterized by widespread inflammation and mediated by autoantibodies against nuclear antigens. Cardiovascular complications are increasingly recognized as major contributors to morbidity and mortality among SLE patients. While accelerated atherosclerosis has traditionally been emphasized as a driver of cardiovascular risk in SLE, emerging evidence also highlights its role in non-atherosclerotic complications such as AAs [71,72,73].

One prominent mechanism linking SLE to aneurysm formation is vasculitis involving medium-to-large arteries. Immune complexes deposited along the endothelium activate complement cascades that recruit neutrophils, macrophages, and lymphocytes into affected vessels. This results in transmural inflammation accompanied by fibrinoid necrosis within the arterial wall—a process that weakens its structural integrity over time [74,75,76].

Additionally, prolonged glucocorticoid therapy—a cornerstone of treatment for SLE—has been implicated as an indirect contributor through its adverse effects on vascular health. Chronic steroid use promotes systemic hypertension while impairing collagen turnover dynamics within connective tissues. Under sustained hemodynamic stress, this combination predisposes patients toward the development of TAAs or AAAs [71,72,74].

Another contributing factor involves antiphospholipid antibodies frequently observed alongside SLE diagnoses. Antiphospholipid syndrome exacerbates the risk of AAs by promoting thrombosis within small or large arteries. This process may trigger localized ischemia leading to secondary arterial remodeling [77].

### 5.2 Rheumatoid Arthritis and Vascular Complications

RA is a chronic autoimmune inflammatory disorder. Although it primarily targets synovial joints, RA has systemic implications, including significant cardiovascular complications. The persistent inflammatory state in RA contributes to both microvascular and macrovascular damage. This can extend to the aortic wall, thereby predisposing patients to the development of AAs.

AA formation in RA is primarily mediated by systemic inflammation, which is characterized by elevated levels of pro-inflammatory cytokines such as TNF-α, interleukin-1 beta (IL-1β), and IL-6. These cytokines perpetuate endothelial dysfunction and activate VSMCs, leading to aberrant remodeling of the ECM through upregulated expression of MMPs. Patients with RA show increased MMP activity, particularly MMP-9. This promotes elastin degradation within the media layer of the aorta, weakening its structural integrity.

Immune cell infiltration into the adventitia of the aorta is another common pathological finding in RA-related vasculopathy. Th1 cells and macrophages release IFN-γ and ROS, further exacerbating oxidative stress and inflammation in the aortic wall. Over time, these processes contribute to vessel dilation and aneurysm formation.

Chronic corticosteroid use is frequently employed in RA management and can also increase susceptibility to AAs by impairing collagen synthesis and promoting vascular fragility. Additionally, RA-associated atherosclerosis driven by systemic inflammation and dyslipidemia may indirectly influence aneurysm development by creating focal areas of hemodynamic stress on the vasculature that is already compromised.

Epidemiological studies suggest that RA patients have elevated risks of both AAAs and TAAs. Regular cardiovascular risk assessment and imaging-based surveillance are crucial for early detection in this high-risk population. Targeted therapies such as TNF inhibitors or IL-6 receptor antagonists may attenuate inflammation-driven vascular damage, although their direct impact on the progression of aneurysms requires further investigation [78,79,80].

### 5.3 Behçet’s Disease and Its Unique Manifestations in the Vasculature

Behçet’s disease is a multisystem inflammatory disorder characterized by recurrent oral and genital ulcers, uveitis, skin lesions, and vasculitis affecting vessels of all sizes [81,82]. Vascular involvement is one of the most severe manifestations of Behçet’s disease, and is often referred to as “vasculo-Behçet syndrome”. Arterial complications include aneurysms, thrombosis, and stenosis; however, arterial aneurysms are of particular concern due to their potential for rupture.

The pathophysiology underlying arterial aneurysm formation in Behçet’s disease involves neutrophil-driven hyperinflammation. Neutrophil infiltration into vessel walls leads to extensive endothelial damage and transmural inflammation. Elevated levels of interleukin-8 (IL-8) drive neutrophil chemotaxis, while the release of ROS by activated neutrophils exacerbates oxidative stress within affected vessels.

The predilection for aneurysm formation at unusual sites such as pulmonary arteries or peripheral arteries is unique to Behçet’s disease. Aortic involvement typically manifests as large fusiform or saccular aneurysms within thoracic or abdominal segments. These lesions are prone to rupture due to extensive destruction of elastic fibers and medial necrosis mediated by immune cell infiltration.

Treatment strategies for Behçet’s-related aneurysms involve aggressive immunosuppression using glucocorticoids, alongside cytotoxic agents like cyclophosphamide, or biologic therapies targeting TNF-α. Endovascular interventions may be necessary for life-threatening complications such as rupture. However, surgical outcomes are often complicated by postoperative thrombotic events or recurrent inflammation, necessitating vigilant follow-up care [83,84,85,86].

### 5.4 IgG4-Related Disease and Thoracic Aortitis

IgG4-related disease is a recently recognized systemic fibro-inflammatory condition characterized by elevated serum IgG4 levels, infiltration of IgG4-positive plasma cells into affected tissues, storiform fibrosis, and obliterative phlebitis. Among its various manifestations is IgG4-related thoracic aortitis—a rare but significant cause of TAA [87].

In IgG4-related thoracic aortitis, chronic low-grade inflammation primarily targets the adventitia but can also extend into the medial layer of the aorta. This process disrupts elastin fibers through inflammatory-mediated remodeling, while inducing fibrotic changes that compromise vessel compliance. Histopathological findings include lymphoplasmacytic infiltrates rich in IgG4-positive plasma cells, accompanied by fibrosis that weakens the vessel wall over time.

The exact etiology of IgG4-related disease remains unclear but likely involves autoimmune mechanisms triggered by genetic predisposition or environmental factors. Aberrant T-helper 2 (Th2) responses are thought to play a key role through cytokines such as IL-4 and IL-10, which promote B-cell activation and IgG4 production [88,89].

Diagnosis of this condition relies on imaging modalities such as contrast-enhanced computed tomography (CT) or magnetic resonance angiography (MRA), which reveal diffuse thickening of the aortic wall, along with laboratory evidence of elevated serum IgG4 levels. Unlike other autoimmune diseases in which systemic markers such as C-reactive protein (CRP) provide an accurate reflection of disease activity, IgG4-related disease often presents without elevated acute-phase reactants despite significant tissue involvement. This diagnostic challenge necessitates histopathological confirmation through the demonstration of hallmark features via biopsy or surgical specimens. Tissue biopsy remains definitive, but is not always feasible given the anatomical constraints.

Treatment involves high-dose glucocorticoids followed by gradual tapering alongside steroid-sparing agents like azathioprine or mycophenolate mofetil for maintenance therapy. In refractory cases or those with extensive vascular involvement posing surgical challenges, biologics like rituximab—a monoclonal antibody targeting CD20 on B-cells—have shown promise in reducing relapse rates while minimizing the dependence on glucocorticoids [90,91,92].

## 6. Diagnosis, Monitoring and Management of Aortic Aneurysms in Patients With Autoimmune Diseases

### 6.1 Imaging Modalities for Detection and Surveillance

The timely diagnosis of AAs in patients with autoimmune diseases requires advanced imaging techniques capable of characterizing both structural abnormalities in the vessel wall and active inflammatory processes that contribute to disease progression.

#### 6.1.1 Ultrasound

Abdominal ultrasound remains an effective first-line tool for screening AAAs due to its accessibility and non-invasiveness.

#### 6.1.2 Computed Tomography Angiography (CTA)

CTA offers high-resolution visualization essential for delineating complex thoracic or abdominal lesions while detecting secondary complications like dissection or rupture.

#### 6.1.3 Magnetic Resonance Angiography

MRA provides superior soft-tissue contrast, enabling assessment beyond simple morphological details. This is critical when evaluating vasculitic components closely linked to autoimmune pathology [15,93,94].

### 6.2 Pharmacological Interventions, Including Immunosuppressive Therapies

Pharmacological management of AAs in patients with autoimmune diseases represents a cornerstone of therapy. The aim is to reduce the inflammatory burden and mitigate disease progression, while minimizing the risks associated with aneurysm rupture or dissection. The therapeutic arsenal includes immunosuppressive agents tailored to the underlying autoimmune condition, as well as adjunct medications targeting vascular health.

#### 6.2.1 Use of Glucocorticoids and Potential Risks

Glucocorticoids are frequently employed as first-line agents in managing autoimmune diseases due to their potent anti-inflammatory and immunosuppressive properties. These drugs inhibit cytokine production, reduce immune cell activation, and stabilize endothelial function—mechanisms that can attenuate inflammation-induced vascular damage contributing to aneurysm formation.

However, the prolonged use of glucocorticoids carries significant risks that must be carefully balanced against their benefits. Chronic glucocorticoid therapy has been implicated in adverse effects such as hypertension, hyperlipidemia, insulin resistance, and impaired collagen synthesis, all of which exacerbate vascular vulnerability. Moreover, long-term glucocorticoid exposure may accelerate degeneration of the aortic wall by disrupting ECM homeostasis. This dual effect necessitates careful dosing strategies and close monitoring of patients receiving such medications.

In patients with active vasculitic processes or other autoimmune conditions contributing to the progression of AAs, short-term glucocorticoid therapy at high doses may be required to control acute inflammation. Subsequently, tapering regimens are implemented to minimize systemic side effects while transitioning patients to maintenance therapies using steroid-sparing agents [15,94].

#### 6.2.2 Role of Biologic Agents in Modulating Disease Activity

The advent of biologic therapies has transformed the management of autoimmune diseases by offering targeted interventions that modulate specific immune pathways implicated in disease pathogenesis. These agents provide an alternative to traditional immunosuppressants for reducing systemic inflammation while preserving vascular integrity.

##### 6.2.2.1 Tumor Necrosis Factor Inhibitors

TNF inhibitors (e.g., infliximab, adalimumab) are commonly used in RA and other inflammatory arthritides and have demonstrated efficacy in reducing systemic inflammation that predisposes to vascular complications [32]. However, their role in directly mitigating the progression of aneurysms remains under investigation.

##### 6.2.2.2 Interleukin-6 Receptor Blockers

IL-6 plays a central role in mediating chronic inflammation associated with RA and giant cell arteritis. Tocilizumab, an IL-6 receptor antagonist, has shown promise in reducing vascular inflammation in these contexts. However, further studies are needed to evaluate its impact on aneurysm outcomes.

##### 6.2.2.3 Rituximab

Rituximab is a B-cell-depleting monoclonal antibody targeting CD20. It is particularly effective for conditions like IgG4-related disease or systemic vasculitis. By suppressing autoreactive B-cell activity and autoantibody production, rituximab helps stabilize inflammatory processes driving aortic wall damage.

##### 6.2.2.4 Other Biologics

Agents targeting IL-17 or interleukin-23 (IL-23) pathways may offer potential benefits in Behçet’s disease or psoriatic arthritis, where Th17-mediated inflammation contributes to the pathogenesis of aneurysms [15,94].

Biologic therapies hold promise for preventing or slowing the progression of aneurysms through precise immune modulation. However, their use must be individualized based on the patient’s specific autoimmune condition, risk profile, and tolerance to treatment. Regular imaging surveillance is essential to assess therapeutic efficacy and to detect any changes in aneurysm size or morphology.

Ongoing clinical trials are currently evaluating metformin [95,96,97] and stem cells [98] as candidate therapeutic strategies for AAA. Previous clinical trials on AAA did not find evidence of benefit from statins [99], antihypertensive drugs [100,101,102], doxycycline [103,104], antiplatelet and anti-thrombotic therapies [105,106], and other drugs with anti-inflammatory activity [107,108].

There are significant limitations to the investigation of targeted therapies, including biologics, for aneurysms. This has prevented definitive evaluation of their effectiveness in halting the progression of aneurysms. Although animal models have shown promise in reducing aneurysm diameter through inhibition of TNF-α [109,110], IL-6 [111,112,113,114], or IL-17 [115,116], these findings have not translated successfully into clinical practice. Possible reasons include poor experimental design, short follow-up periods, and small cohort sizes [117,118,119,120].

### 6.3 Future Therapeutic Strategies

Current research in the field of AA treatment focuses on modulating key pathophysiological mechanisms involved in AA development and progression, such as inflammation, extracellular matrix degradation, loss of VSMCs, oxidative stress, mitochondrial dysfunction, endoplasmic reticulum stress and regulation of epigenetic changes [108], thus primarily slowing aneurysm progression.

Further, regenerative therapies, potentially reversing vascular degeneration, have been studied; with eventually benefit of reducing the requirement for surgery [121]. First, cell-based approaches using VSMCs, endothelial cells, or mesenchymal stem cells, delivered either intravascularly or periadventitially, support AA repair through tissue regeneration, immunomodulation, and paracrine signaling. This results in reduced inflammation and matrix degradation, while elastin restoration and endothelial repair is promoted [121,122,123]. Alternatively, cell-free therapies—including extracellular vesicles, mitochondrial transfer, gene therapy, targeted drug delivery systems, and biomaterials—have been also proposed to reproduce the regenerative effects of cells [121].

## 7. Prognosis and Outcomes for Patients With Concurrent Autoimmune Disorders and Aortic Pathologies

The prognosis of patients with concurrent autoimmune disease and AA depends on multiple factors, including disease activity, adherence to treatment protocols, timely detection of vascular complications, and access to multidisciplinary care. Patients managed appropriately with combined pharmacological therapy to control systemic inflammation, and with surgical interventions when indicated, generally have improved survival rates compared to untreated counterparts.

However, long-term outcomes remain variable due to the persistent risk of recurrent vascular events driven by ongoing immune dysregulation, despite therapeutic efforts. Regular monitoring using imaging modalities such as CTA or MRA allows early identification of progressive changes that warrant prompt intervention.

Advances in biologic therapies hold promise not only for controlling autoimmune activity, but also for improving vascular outcomes. However, further clinical trials are needed to establish definitive links between targeted immune modulation and reduced aneurysm progression across diverse patient populations [94].

## 8. Conclusions and Future Perspectives

Autoimmune diseases play a pivotal role in the development and progression of AAs through mechanisms such as chronic inflammation, immune-mediated vascular remodeling, and the contribution of genetic predisposition and/or variation. Recognizing these connections is critical for improving early detection, risk stratification, and targeted treatment strategies for patients in whom aneurysms evolve in the context of autoimmune conditions. Future research should therefore be directed towards the discovery of biomarkers that can help to identify patients at risk of developing AAs. Besides biomarkers present in the circulation (e.g., cytokines, MMPs, miRNAs, etc.), relevant candidates for future research include genetic biomarkers. These can be either panels of multiple polymorphisms reflecting the polygenic nature of chronic inflammation and vascular remodeling, or individual variants that underlie genetic syndromes encompassing aneurysms. The combination of several circulatory biomarkers with genetic profiling could result in increased sensitivity and specificity for the early detection of AAs.

Besides diagnostic-directed research, other priorities lie in the development of novel treatments. Considering the shared pathogenetic mechanisms described earlier between autoimmunity and aneurysms, the search for new treatments should involve novel inflammatory regulators, inhibitors, and/or molecules aimed at epigenetic mechanisms. Finally, it remains to be determined whether recent experimental therapies for autoimmune diseases, such as chimeric antigen receptor T cells (CAR-T cells) and/or bispecific antibodies [124], could also be effective against aneurysms in the autoimmune context.

Continued interdisciplinary research involving well-defined patient groups sourced from multiple centers and unifying relevant biomedical specialties is essential for uncovering novel insights and optimizing diagnostic and therapeutic approaches. This focus should ultimately reduce the morbidity and mortality from aneurysms in the context of autoimmune disease, as well as clarify the complex interplay between immunology, genetics, and vascular pathology.
